# Optimising reproductive and child health outcomes by building evidence-based research and practice in South East Asia (SEA-ORCHID): study protocol

**DOI:** 10.1186/1471-2288-7-43

**Published:** 2007-09-24

**Authors:** David J Henderson-Smart, Pisake Lumbiganon, Mario R Festin, Jacqueline J Ho, Hakimi Mohammad, Steve J McDonald, Sally Green, Caroline A Crowther, the SEA-ORCHID Study Group

**Affiliations:** 1 NSW Centre for Perinatal Health Services Research, Queen Elizabeth ll Research Institute, University of Sydney, NSW 2006, Australia; 2 Department of Obstetrics and Gynaecology, Khon Kaen University, Khon Kaen 40002, Thailand; 3 Department of Obstetrics and Gynecology, College of Medicine, University of the Philippines Manila, Manila 1000, Philippines; 4 Department of Paediatrics, Penang Medical College, Penang 10450, Malaysia; 5 Department of Obstetrics and Gynecology, Gadjah Mada University, Yogyakarta 55281, Indonesia; 6 Australasian Cochrane Centre, Monash University, Melbourne, VIC 3168, Australia; 7 Discipline of Obstetrics and Gynaecology, University of Adelaide, SA 5006, Australia

## Abstract

**Background:**

Disorders related to pregnancy and childbirth are a major health issue in South East Asia. They represent one of the biggest health risk differentials between the developed and developing world. Our broad research question is: Can the health of mothers and babies in Thailand, Indonesia, the Philippines and Malaysia be improved by increasing the local capacity for the synthesis of research, implementation of effective interventions, and identification of gaps in knowledge needing further research?

**Methods/Design:**

The project is a before-after study which planned to benefit from and extend existing regional and international networks. Over five years the project was designed to comprise five phases; pre-study, pre-intervention, intervention, outcome assessment and reporting/dissemination. The study was proposed to be conducted across seven project nodes: four in South East Asia and three in Australia. Each South East Asian study node was planned to be established within an existing department of obstetrics and gynaecology or neonatology and was intended to form the project coordinating centre and focus for evidence-based practice activities within that region. Nine hospitals in South East Asia planned to participate, representing a range of clinical settings. The three project nodes in Australia were intended to provide project support.

The intervention was planned to consist of capacity-strengthening activities targeted at three groups: generators of evidence, users of evidence and teachers of evidence. The primary outcome was established as changes in adherence to recommended clinical practices from baseline to completion of the project and impact on health outcomes.

**Discussion:**

The SEA-ORCHID project was intended to improve care during pregnancy and the perinatal period of mothers and their babies in South East Asia. The possible benefits extend beyond this however, as at the end of this project there is hoped to be an existing network of South East Asian researchers and health care providers with the capacity to generalise this model to other health priority areas. It is anticipated that this project facilitate ongoing development of evidence-based practice and policy in South East Asia through attracting long-term funding, expansion into other hospitals and community-based care and the establishment of nodes in other countries.

## Background

### Importance of the health problem

Each year there are over half a million maternal deaths worldwide and 98 percent of these occur in the developing world. This represents a tragic and striking health risk differential between the developed and developing world. For women in Asia the lifetime risk of maternal death is one in 65 compared with one in 1,800 for women in developed countries [1].

The main causes of mortality directly related to pregnancy and childbirth are unsafe abortions, bleeding, infection, hypertension (including eclampsia) and obstructed labour [2]. The majority of maternal deaths occur after the birth but many are related to conditions that present earlier in pregnancy. Serious acute and chronic maternal morbidity has been estimated to occur in one in four women [3].

Perinatal and early infant deaths are also high in developing countries. Each year there are almost eight million stillbirths and early neonatal deaths. These arise because of poor maternal health and pregnancy care. Associated morbidity manifests as low birth weight, asphyxia and infection [4].

The high rate of maternal, infant and child mortality in SE Asian countries has been associated with poverty, reduced education and literacy, lack of remunerative employment, low social status, and limited access to health services and family planning [5]. SE Asia accounts for one quarter of the world's population and more than half of the worlds poor. Solutions to these problems require an inter-sectoral approach. This has been supported by many international bodies such as the World Health Organization, World Bank, United Nations Children's Fund and United Nations Population Fund.

Access to scientifically valid and up-to-date information is a prerequisite for allocation of resources according to evidence and need. Most health workers and policy-makers in developing countries do not have easy access to the latest reliable information on effective care or may not have the skills to evaluate and implement such evidence [6].

A variety of problems arise when clinical practices that are not based on sound scientific evidence are incorporated into established healthcare practice. Valuable resources continue to be used in some developing countries to fund practices of unknown effectiveness, for example electronic fetal monitoring during labour [7]. On the other hand, interventions that have been shown to be both cheap and effective, such as magnesium sulphate to prevent and treat eclampsia, have not been widely implemented [8]. In an empirical example of clinical practice being at odds with published recommendations, a study conducted in six centres in SE Asia and two in the United States of America, demonstrated a large variation in the use of antibiotic prophylaxis in caesarean section, despite there being strong evidence supporting its use [9]. Only two of the eight participating centres routinely administered appropriate regimens of antibiotics at the appropriate time.

Clearly a mechanism is needed to provide healthcare practitioners in the developing world with access to relevant evidence, a means of determining their own evidence requirements and the infrastructure to disseminate and implement clinical practice change based on that evidence. The effects of this provision then need to be evaluated to inform future initiatives aiming to improve health care.

### Previous work instigating this project

The Cochrane Collaboration is an international organisation of healthcare practitioners, researchers and consumers that prepares and disseminates systematic reviews of high quality evidence about the effects of healthcare interventions [10]. The growth of the Cochrane Collaboration is well known, with over 3000 reviews published since 1995 and an estimated 11,500 contributors worldwide.

However, this growth has been predominantly in the developed world, and a key objective of the Cochrane Collaboration is to ensure growth of this research activity in the developing world. The Cochrane Developing Countries Network has been established to pursue strategies for encouraging the involvement of contributors from low- and middle-income countries, both in producing reviews and in implementing their findings. In 2004, the level of developing country involvement in the Cochrane Pregnancy and Childbirth Group was modest, with 59 review authors from 11 developing countries and only eight review authors from SE Asia. In the Cochrane Neonatal Group there were 11 review authors from seven developing countries and only three of these were from SE Asia.

In a joint project between WHO, UNDP/UNFPA/WHO/World Bank Special Programme of Research Development and Research Training in Human Reproduction (HRP), South African Cochrane Centre and WHO Regional Office for Africa, the *WHO Reproductive Health Library *[11] derived in part from reviews in *The Cochrane Library*, was made available and practitioners trained in its use within South Africa [12]. Introducing a similar initiative in SE Asia, along with increasing the capacity for involvement in the Cochrane Collaboration and use of *The Cochrane Library*, has the potential to contribute to addressing the issues surrounding maternal mortality and morbidity.

Our project, which began in 2004, planned to build on these previous strategies and go beyond the implementation of evidence alone to assess the effect of a more far-reaching intervention of identifying evidence needs; training and support in evidence generation, synthesis and use; and provision of research infrastructure to facilitate these evidence-based practice activities.

### Rationale for this project

It has been stated that providing access to reliable health information for workers in developing countries is potentially the single most cost effective and achievable strategy for sustainable improvement in health care [13]. Information provision alone however is not enough: we need to ensure that clinical practice changes in response to that information. While little is known about the best ways to change the behaviour of healthcare workers and so to implement available evidence, we do know that it is a complex process requiring access to information, skills to interpret that information and a sense of having contributed to the process. The Cochrane Collaboration has had success in the developed world in involving clinicians in the process of generating, synthesising and using evidence. The intervention in this project will draw on the experience of the past 10 years in building the Cochrane Collaboration through infrastructure provision, training, support and methodological development and will involve local researchers to ensure the intervention is regionally appropriate and designed to increase capacity within SE Asia.

In order to increase the uptake of effective treatments and stop the use of harmful ones, it is essential that healthcare communities within SE Asia are the drivers of the project. This includes promoting interventions that are locally appropriate, identifying important research questions, conducting relevant research and evidence synthesis, and training local practitioners in the implementation of research findings. It has been demonstrated that a sense of local ownership of projects and processes aiming to improve practice, including evidence generation, contributes to the success of implementing evidence [14].

In 2001, the Thai Cochrane Network, based at Khon Kaen University, became the first registered group of the Cochrane Collaboration in SE Asia and provides the ideal platform from which to launch this current initiative. To maximise the potential of this intervention we plan to build upon existing partnerships and organisations to extend current capacity [15].

### Aims and objectives

The project was designed to address the following broad scientific question: Can the health of mothers and babies in Thailand, Indonesia, the Philippines and Malaysia be improved by increasing capacity for the synthesis of research, implementation of effective interventions, and identification of gaps in knowledge needing further research in those countries? The objectives of the project were intended to answer the following questions as components of the broad question:

1. What is the current teaching and practice related to pregnancy and childbirth in SE Asia?

2. What are the local barriers to the use of research in SE Asia and how can these barriers be overcome?

3. Will targeted interventions to build capacity for the generation, evaluation, synthesis and implementation of relevant evidence lead to improved research output, research implementation and better health outcomes for women and babies in SE Asia?

## Methods/Design

The SEA-ORCHID project was designed as a before-after study, using action research to design and implement the intervention. It set out to extend and benefit from existing networks. The project was planned to be conducted across seven nodes, four in SE Asia and three in Australia (Table 1). Each SE Asian study node was planned to be established within an existing department of obstetrics and gynaecology or neonatology and was intended to form both the project co-ordination centre and the focus for evidence-based practice activities within the region.

**Table 1:** SEA-ORCHID project nodes and participating SE Asian hospitals

**Country**	**Node**	**South East Asian Hospitals**	**Investigator**
**Thailand**	Department of Obstetrics and Gynaecology, Khon Kaen University, Khon Kaen	Srinagarind Khon Kaen University Hospital Khon Kaen Regional Hospital Kalasin General Hospital	Pisake Lumbiganon
**Philippines**	Department of Obstetrics and Gynecology, College of Medicine, University of the Philippines Manila, Manila	Philippine General Hospital (University of the Philippines) Dr. Jose Fabella Memorial Hospital	Mario Festin
**Malaysia**	Department of Paediatrics, Royal College of Medicine, Ipoh	Ipoh Hospital, Perak Universiti Sains Malaysia, Kota Bharu	Jacqueline Ho
**Indonesia**	Department of Obstetrics and Gynecology, Gadjah Mada University, Yogyakarta	Dr. Sardjito Hospital Sleman District Hospital	Mohammad Hakimi
**Australia**	NSW Centre for Perinatal Health Services Research, Sydney		David Henderson-Smart
**Australia**	Australasian Cochrane Centre, Monash University, Melbourne		Sally Green
**Australia**	Australian Research Centre for the Health of Women and Babies, Discipline of Obstetrics and Gynaecology, University of Adelaide, Adelaide		Caroline Crowther

Activities and timelines for each project phase are outlined in the following sections and summarised in Table 2. A SEA-ORCHID project meeting was planned to be held annually to review progress and strengthen regional collaborative networks, and was intended to be timed to coincide with a local event (eg. perinatal meeting, hospital seminar) to enhance promotional opportunities and maximise the benefits of having several international speakers present.

**Table 2:** SEA-ORCHID project plan and activities associated with each phase

	**Year**	**Australian Support Centres**	**SE Asian Study Nodes**
**Pre Study Phase**	**2004**	*First SEA-ORCHID project meeting Malaysia: focus on planning, and development of assessment tools*	
		Recruit project manager: five year appointment Recruit project administrator five-year appointment Develop data extraction and recording tools for primary and secondary outcomes Set up and pilot web-based forms and data entry system Obtain ethics committee approval	Recruit project supervisor (Thailand): five year appointment Recruit project administrator (Thailand): five year appointment Set up project nodes Obtain ethics committee approval from local institutions Begin promotion of project within SE Asian nodes Recruit and train four SE Asian fieldworkers

**Pre-intervention**	**2005**	Data cleaning and ongoing analysis from web-based system as data entered from nodes Recruit and train three educators/systematic reviewers/guideline developers (one at each support centre): three year appointment Host training of SE Asian educators Develop training materials Measure baseline involvement of SE Asia in Cochrane Collaboration	Carry out baseline data collection of primary and secondary outcomes (data entry via web) Recruit and train nine educators/guideline developers/systematic reviewers from participating hospitals Plan and organise training events and associated local promotion Advertise and select eight fellows for Australian fellowships in 2006
		*Second SEA-ORCHID project meeting the Philippines: review and report findings from baseline data collection and adapt intervention to ensure it addresses identified barriers to change*	
		Conduct teaching tour	

**Intervention**	**2006 to mid 2007**	Ongoing support for training in systematic reviews and EBP Partner in guideline development Host eight SE Asian fellowships Support SE Asian nodes in all activities	Conduct training in systematic reviewing and EBP and undertake systematic reviews Develop guidelines Disseminate the evidence from Cochrane reviews Identify local priorities for reviews
		*Third SEA-ORCHID project meeting Thailand: focus on developing undergraduate programs and planning for funding beyond project to sustain nodes*	
		Two teaching tours (one adjacent to SEA-ORCHID project conference)	

**Outcome Assessment**	**mid 2007 to mid 2008**	Provide all data extraction and recording tools Outcome assessment of SE Asian involvement in Cochrane Collaboration Data monitoring, cleaning and analysis	Recruit and train four fieldworkers for outcome assessment Complete collection of primary and secondary outcome data (data entry via web)
		*Fourth SEA-ORCHID project meeting Indonesia: review outcome assessment, plan for activities beyond employment of trainers*	

**Report**	**End 2008**	*Fifth SEA-ORCHID project meeting (investigators) interpret analyses, plan final reports and assist with planning of future initiatives*	
		Write up of final reports and associated publications	

### Pre study phase (2004)

During this phase of the study, we planned to establish three Australian support nodes and a project node within each of the four SE Asian participating countries. Under the supervision of the local SEA-ORCHID investigator, each node put in place the infrastructure to support and sustain the project. This involved purchasing equipment, employing and training staff, and seeking local ethics approval. As part of efforts to promote the project we set up a dedicated website  that includes information about the project; a library of project materials, resources and presentations; and a secure data-entry system.

#### First SEA-ORCHID project meeting

The investigators planned to meet in Malaysia before the start of the pre-intervention phase to ensure all nodes were functioning smoothly, and to plan the development of the data collection assessment tools, the training of local fieldworkers in web-based data entry and finalise project procedures.

### Pre-intervention phase (2005)

The principal activities during this phase were the collection of baseline data and the recruitment and training of staff who were delivering the intervention. Three categories of information were planned to be collected at baseline and post-intervention:

#### 1. Adherence to recommended clinical practices and health outcomes (primary outcomes)

We planned to assess whether current clinical practice during pregnancy and childbirth follows best-practice recommendations, and assess the impact of some these practices on the health of mothers and babies. Adherence to 12 areas of current recommended clinical practice and 13 health outcomes of mothers and babies in the four SE Asian centres were the primary outcome. The various practices and their associated health outcomes were selected on the basis of clear evidence from Cochrane systematic reviews (Table 3).

**Table 3:** Health-related outcomes

**Recommended practice**	**Outcome intended to reduce**
**Beneficial forms of care**	
Antibiotics for preterm prelabour rupture of membranes (pPROM)^16^	Chorioamnionitis; neonatal sepsis
Corticosteroids prior to preterm birth^17^	Neonatal death; complications of preterm birth
External cephalic version for breech presentation at term^18^	Caesarean section rate; birth trauma
Continuous support during labour^19^	Caesarean section rate
Magnesium sulphate for eclampsia and pre-eclampsia^20,21,22^	Maternal death; eclampsia
Active management of third stage of labour^23^	Postpartum haemorrhage; maternal death
Intraoperative antibiotics during caesarean section^24^	Maternal infection
Vacuum extraction (versus forceps) for operative delivery^25^	Perineal injury; postpartum haemorrhage
Immunisation for Hepatitis B^26^	Hepatitis B infection

**Forms of care likely to be harmful**	
Routine episiotomy^27^	Perineal injury; maternal infection
Routine shaving* ^28^	Maternal infection
Routine enemas* ^29^	Maternal infection

* No clear evidence from Cochrane reviews to support or refute use, but identified as practices of importance to research and evaluate

Data related to the primary outcome were planned to be collected by field-workers over a nine-month period from the consecutive case reports of 1000 women admitted to each of the participating SE Asian hospitals. Specially designed data collection forms were designed to ensure the information was extracted regarding the use of recommended practices, if applicable, together with the subsequent health outcomes of the mother and baby.

#### 2. Current involvement in evidence-based practice (secondary outcome)

We planned to determine the level of activity and involvement in generating, teaching and using evidence at each SE Asian node by:

- identifying current research projects relevant to pregnancy and childbirth (from government funding bodies, ethics committees and research registers)

- identifying local clinical practice guidelines related to pregnancy, childbirth and infant care (through a survey of health departments and clinical associations)

- assessing the amount of undergraduate medical teaching related to evidence-based practice (through a survey of medical schools)

In addition, we planned to assess the contribution of SE Asians to the Cochrane Collaboration with respect to numbers of reviews and contributors (through a search of *The Cochrane Library*).

#### 3. Potential local barriers to practice change (secondary outcome)

The investigators and fieldworkers planned to carry out a series of surveys and interviews within the participating institutions to establish current knowledge, attitudes and extent of evidence-based practice and the factors specific to that node which may form a barrier to practice change, or may be used to enhance practice change. Specific knowledge and beliefs about evidence-based practice, research results and systematic reviews were assessed, along with perceived difficulties in accessing, appraising and using research-based information. Culturally specific barriers to the use of the selected pregnancy and childbirth healthcare practices were explored, and any relevant issues used to modify the intervention.

#### Data management and analysis

A customised, secure database was designed to be housed on the project website to allow fieldworkers to enter the extracted information on data collection forms directly into the database . The analysis plan for the baseline data included descriptive statistics to provide a picture of current clinical practices and health outcomes in the management of pregnancy and childbirth at the four SE Asia nodes. Continuous variables were to be presented as means and standard deviations (or medians and ranges if data are not normally distributed) and dichotomous variables presented as numbers and frequencies of events.

#### Preparing for the intervention

Twelve educational training lecturers (educators), nine from the SE Asian participating hospitals and one at each of the Australian support nodes were recruited and trained, and materials developed for use in the training components of the intervention. A fellowships program was planned to enable health professionals and researchers from SE Asia to visit Australia to receive advanced training in the skills of evidence-based practice, guideline development, critical appraisal and systematic reviewing. These fellowships were advertised in the four SE Asian countries, and the recipients selected by the SE Asian investigators.

#### Second SEA-ORCHID project meeting

The investigators and educational training lecturers planned to meet in the Philippines towards the end of the pre-intervention phase to review baseline findings and to finalise the intervention, taking into consideration the barriers to practice change identified by the baseline questionnaires and interviews. Following this meeting, the Australian-based educational trainers planned to carry out the first SE Asia teaching tour, conducting training events with the local training lecturer. This was the beginning of the intervention phase.

### Intervention phase (2006 – mid 2007)

The planned intervention can be divided into the following categories with the timing of delivery according to the project schema in Table 2:

#### i) Training

The provision of training to support various evidence-based practice initiatives is a major component of the intervention being tested. Local researchers and clinicians planned to conduct training with the support of the Australian-based project members through the provision of materials and teaching assistance. Training will comprise of a fellowships program, teaching tours of all four SE Asian nodes by the Australian-based trainers in partnership with local trainers, and five project meetings. Activities will focus around three core groups:

#### 1. Generators of evidence and evidence-based materials

The emphasis is on setting up training programs in critical appraisal, systematic reviewing and guideline development. The Cochrane Collaboration takes great pride in the quality and relevance of its training programs, and the Australian investigators have all been involved in the development and delivery of training in evidence-based practice for many years. We have access to an array of training methods and tools that have been tried and tested cross-culturally and in a variety of formats.

#### 2. Users of the evidence: clinicians and policy makers

We planned to conduct training for clinicians in using *The Cochrane Library *and *WHO Reproductive Health Library*; implementing and using guidelines; and accessing and interpreting evidence. Training will be open to all disciplines involved in the management of the mothers and babies unit (ie. medicine, nursing, community health workers). For policy makers we plan to conduct tailored training workshops around understanding and interpreting evidence. These will be adapted from a series of workshops for policy makers developed by the Australasian Cochrane Centre and will be taught in partnership with SE Asian contributors.

#### 3. Educators about evidence: teachers and trainers

To build capacity and to ensure the outcomes from this project are sustained and extended to other clinical areas, we planned to conduct training events targeting future clinical trainers and potential opinion leaders in SE Asia. In addition, to facilitate practice change through investment in our future clinicians, we plan to provide material and training on the principles of evidence-based practice to those involved in undergraduate programs for doctors, nurses and allied health workers. We will achieve this through existing networks linking the investigators and medical educators in SE Asia.

#### ii) Systematic reviewing

We planned to work with those providing care to women and babies in SE Asia to identify important questions for which systematic review evidence is lacking. Interventions appraised in systematic reviews were clinically relevant and culturally acceptable for the management of pregnancy and childbirth in SE Asia. These reviews were intended to be prepared with support from investigators and educators and published in *The Cochrane Library*, and the results actively disseminated to clinicians through guidelines, training events and other publications.

#### iii) Guideline development

Based on experience with developing guidelines for the Australian National Health and Medical Research Council, we planned to co-ordinate and facilitate locally relevant evidence-based guideline development and implementation.

#### iv) Infrastructure support

Health care based on evidence requires not only trained personnel, but also physical infrastructure, for example, computers and access to information and support. This project was designed to improve research infrastructure and capacity within SE Asia to address locally relevant and culturally specific questions by providing a central focus for research activity and skills.

#### v) Academic exchange

Over the course of this project there were numerous opportunities for academic exchange. Fellowships in Australia were offered to researchers and clinicians from SE Asia, and the Australian-based educators will travel to all the nodes to conduct workshops and partner the SE Asian educational trainers in the development of materials.

#### vi) Promotion

The five project meetings were planned to be held to coincide with local clinical meetings and events. This allowed access to the SE Asian and Australian investigators as speakers at these events and assist in the promotion of evidence-based practice, the SEA-ORCHID project and the results of the research conducted. In addition, the work resulting from the project was intended to be published in the academic literature and presented at local, national and international perinatal meetings and Cochrane Colloquia.

#### vii) Input into the undergraduate curriculum

We planned to facilitate teaching of evidence-based practice skills in medical, nursing and allied health schools by sharing knowledge, skills and materials from Australia together with content relevant to SE Asia. This was intended to be facilitated based on the audit of evidence-based practice learning gathered as part of the baseline data collection.

#### Third SEA-ORCHID project meeting

A larger meeting involving the investigators, educational trainers and SEA-ORCHID fellows was planned to be held in Thailand. This meeting intended to review progress with the intervention, identify potential future research projects based on identified research needs, and consider opportunities for funding beyond the project to sustain the SE Asian nodes and evidence-based practice activity. Again, immediately following the meeting, Australian educational trainers planned to undertake a tour to support local educators in training events.

### Outcome assessment phase (mid 2007 – mid 2008)

The methodology of outcome assessment was designed to duplicate that of the baseline data collection, with SE Asian fieldworkers recruited and trained to carry out the tasks overseen by the local investigators, and facilitated through a web-based data collection system.

#### Primary outcome

The primary indication of the value of the intervention was planned as changes in the process of care during pregnancy and childbirth and any associated impact on health outcomes for mothers and babies. Adherence to recommended practice and maternal/neonatal outcomes (Table 3) were recorded from consecutive case records (1000 per participating hospital) over a nine-month period in a similar fashion to that of the baseline data collection. Descriptive statistics were intended to be used to summarise the data and changes from baseline to endpoint calculated for all measures in an effort to demonstrate practice change and improvement in health.

#### Secondary outcomes

The following activities were planned to be re-assessed in a similar way as at baseline and compared:

a) Involvement in evidence-based practice activity

b) Knowledge and beliefs and the potential barriers to practice change

c) SE Asian contribution to the work of the Cochrane Collaboration

In addition, sustainability of activities beyond the project through procurement of future research funding was intended to form a measure of the impact of the intervention.

#### Fourth SEA-ORCHID project meeting

The investigators and trainers planned to meet in Indonesia to review the intervention phase and ensure the project teams are prepared for the post-intervention data collection. There was also planned to be a focus on sustaining the activities initiated during the SEA-ORCHID project once funding ends, and planning for future intra- and inter-regional collaborations and partnerships.

### Reporting phase (mid 2008)

Dissemination of results will be an integral feature of the project and we anticipate the following publications:

- management of pregnancy and childbirth in SE Asia prior to the intervention

- perceived barriers to clinical practice change in SE Asia

- several systematic reviews and clinical practice guidelines relevant to the management of pregnancy and childbirth in SE Asia and other resource poor settings

- experiences of training in partnership across cultures

- final report and publications regarding the effect of the intervention

#### Fifth SEA-ORCHID project meeting

The final conference will be attended by the investigators and will focus on interpretation of the analysed results, planning final reports and associated publications, and planning future work in SE Asia.

## Discussion/Conclusion

Basing healthcare practice and policy on evidence ensures the maximal benefit for investment. This is important in all communities but particularly when resources are low and the threats to health are large. This project has the potential to impact significantly on the health of mothers and babies in SE Asia by improving care during pregnancy and birth. Resulting from this project, we anticipate that within SE Asia there will be enhanced capacity to:

• train existing and future clinicians to interpret and implement evidence

• ensure locally relevant evidence is available and accessible

• identify important questions yet to be answered and so take a cost effective and high impact approach to future research funding

• base policy decisions on research findings

• develop and implement local clinical practice guidelines

• contribute more relevant systematic reviews for Cochrane Library

The possible benefits, however, extend beyond maternal and newborn care as at the end of this project there will be an existing network of SE Asian researchers and clinicians with the capacity to generalise this model to other health priority areas. It is anticipated that this project will facilitate the ongoing development of evidence-based practice and policy in SE Asia through attracting additional long term funding, expansion into community-based care and the establishment of nodes in other countries in the region.

## Abbreviations

SE Asia – South East Asia

SEA-ORCHID – South East Asia Optimising Reproductive and Child Health in Developing Countries

UNDP – United Nations Development Programme

UNFPA – United Nations Population Fund

UNICEF – United Nations Children's Fund

WHO – World Health Organization

## Competing interests

The author(s) declare that they have no competing interests.

## Authors' contributions

DH-S, PL, SG and CC conceived the study and participated in the project design. All authors were involved in the development of the design of the project, drafting of the manuscript and revising it critically for important intellectual content and have given final approval of the version to be published.

## Pre-publication history

The pre-publication history for this paper can be accessed here:


